# Tooth Loss Condition and Social Discrimination in Brazilian Healthcare Services

**DOI:** 10.3389/ijph.2021.586597

**Published:** 2021-03-12

**Authors:** Alexandre F. Bulgarelli, Camila M. dos Santos, Rafaela S. Rech, Alexandre Baumgarten, Bárbara N. Goulart

**Affiliations:** ^1^ Graduate Program in Collective Health, Federal University of Rio Grande do Sul, Porto Alegre, Brazil; ^2^ Department of Preventive and Social Dentistry, Faculty of Dentistry, Federal University of Rio Grande do Sul, Porto Alegre, Brazil; ^3^ Psychology Institute, Department of health and human communication, Universidade Federal do Rio Grande do Sul, Porto Alegre, Brazil

**Keywords:** prejudice, health services, cross-sectional studies, esthetics, dental, oral health, dental public health

## Abstract

**Objective:** To explore factors associated with social discrimination against users of health services regarding dental aesthetic conditions.

**Methods:** Based on a Brazilian National Survey, multivariate Poisson regressions with robust variance were used to explore the association of outcome discrimination related to different motivations in health services and exposure to sociodemographic and dental variables. Effect modification by complete prosthesis wearing was assessed.

**Results:** Among the 60,200 people interviewed, 11.5% reported being discriminated against in health services. For women, a higher prevalence of discrimination was found among those in the age group of 30–44 years. For both sexes, discrimination was associated with black and brown skin color. Regarding dental characteristics, the higher the tooth loss was, the higher the prevalence of discrimination; however, complete prosthesis wearing presented as a protective factor. Social discrimination was the major motivation for reported discrimination and presented higher prevalence in edentulous individuals who did not wear prosthesis.

**Conclusion:** Dental loss may lead to self-reported discrimination in health care services. The prevalence of discrimination increases when tooth loss increases, and the major reason associated is social discrimination.

## Introduction

Worldwide, people face multiple forms of discrimination and prejudice, and the discrimination users of public healthcare services have been facing recently represents an important concern (1, 2). This phenomenon can be understood as exposure to a social experience that leads a person to sense a discriminatory effect and face stressor conditions (1). Furthermore, social discrimination may be a trigger point for judgment through stereotyping, leading to individualization, prejudice and segregation (1).

These factors are important concerns to be analyzed, particularly for individuals who are already vulnerable, such as dependent older persons and those who need healthcare (2–4). Social discrimination can be perceived as any manifestation of prejudice regarding an explicit dissociation of judgment or an implicit prejudice towards someone (5). Such prejudice, found worldwide, is usually expressed through consultation delays, negligence of communication, refusal of treatment, unfriendly attitude towards a patient, and even harassment (5). Unequal health treatment regarding racial and ethnic conditions is a manifestation of discrimination. Some health systems, healthcare providers and healthcare plan managers contribute to this form of discrimination (6). In addition, implicit discrimination can be manifested by pejorative ways of communication or non-verbal behavior, such as no eye contact or physical proximity during patient assistance and consultation (4). The perceptions of discrimination regarding healthcare assistance might be different in diverse cultures and countries. There is a consensus that stereotyping by health services is an attitude of discrimination in any culture and is unacceptable (6).

Determinants of social discrimination during healthcare assistance may be associated with a variety of situations regarding patient characteristics, such as economic vulnerability, obesity, skin color, sex, age, poor dental appearance, and sexual orientation (2, 4, 7, 8). All these assumptions reflect patients’ negative expectations towards healthcare assistance, represent discriminatory health care treatment and have a long-term impact on patients’ health (9, 10). Understanding the association between health care utilization and possible discrimination at any health service is important to support the design of policies focusing on disparities in health services access (11).

Dental aesthetic conditions and user appearance may lead to discrimination in healthcare services (12). Even though it is a particularly important concern to be discussed, studies regarding self-reported discrimination in healthcare services and patient dental aesthetic conditions are still scarce (2, 3, 8, 12). Studies report that poor oral health conditions are commonly perceived as a problem for vulnerable people. Poverty, socioeconomic disadvantages, race, and social disparities suggest a potential for discrimination regarding dental appearance and conditions and access to healthcare services (8, 13–15). Furthermore, having bad teeth or a missing tooth can be a stressor condition regarding social discrimination. For instance, older adults with severe tooth loss have lack of functional dentition (16), and this can be understood as a condition of vulnerability. The social perception of a bad dental appearance leads a person with a missing tooth to experience negative feelings about himself or herself, and this may cause psychological distress (8). This is an important issue from a clinical perspective. In regard to this concern from a clinical perspective, patients with chronic conditions who experience a discriminatory situation reduce their engagement with the healthcare system (17).

Under this background, the research question of the present study is the following: Are patients’ dental aesthetic conditions, such as lack of teeth and use of a prosthesis, possibly associated with social discrimination in healthcare services? This research question was enriched by another question: Do patient characteristics, such as sex, social vulnerability and age, motivate discrimination in a health services? To investigate these issues, the present study aims to analyze social discrimination in terms of the motivation perceived by users of health services in Brazil regarding dental aesthetic conditions.

## Methods

### Data Collection and Study Sample

This is a population-based cross-sectional study, developed to analyze self-reported positive discrimination and based on data from an epidemiological household survey across Brazil. This study used part of the data collected by the Brazilian National Health Survey (PNS) that was conducted between August 2013 and February 2014 by the Ministry of Health in partnership with the Brazilian Institute of Geography and Statistics (IBGE). The PNS was specifically designed to gather information on several aspects of health (18). Since this study uses self-reported data, the PNS was supported by a Patient-Reported Outcomes Measures (PROM) perspective and properly assembled instruments. This allowed the generation of data providing information on patient satisfaction, the impact of received treatment and reliable patient perceptions (19, 20).

The PNS sample design was organized using multi-stage clustering. In each stage, units were selected by simple random sampling. In the first stage, the census tracts were the primary sampling units. Special census tracts (barracks, military bases, lodges, camps, boats, penitentiaries, penal colonies, prisons, chains, asylums, orphanages, convents, hospitals, indigenous villages and census tracts located in indigenous lands) were excluded from the survey. In the second stage, 10 to 14 households were selected in each primary sampling unit. Lastly, in the third selection stage, one resident aged 18 years or over, and able to respond, was selected.

To calculate the sample size of the PNS, the following aspects were considered: estimation of proportions with the desired level of precision in 95% confidence intervals; the effect of the sampling plan, which was multi-stage clustered sampling; the number of households selected in each census tract; and the proportion of households with people in the age group of interest. The sample size calculation resulted in 79,875 households. Mean values, variances, and sample design effects were taken into consideration in the calculation, predicting a non-response rate of 20%. The following losses were considered: closed or empty domicile; the refusal of the residents to respond; and inability to interview the resident after three or more attempts, even with scheduled visits. Given that the PNS has a complex sampling design and unequal selection probabilities, sampling weights for households and their residents were calculated as the product of the weight of the corresponding primary sampling unit and the inverse of the probability of selection of the household within the primary sampling unit. The researchers adjusted the weights to correct for non-response and to calibrate the estimates to total populations known from other sources. The final weight of the selected residents was calculated as the product of the weight of the household and the number of eligible residents in the household. Details of the sampling plan and of other methodological aspects have been published elsewhere (21). This study analyzed the information of 60,200 individuals aged between 18 and 65 years.

For data collection, external evaluators were selected and uniformly trained by means of a field manual. The data collection was carried out between August 2013 and February 2014, using personal digital assistants, each containing an app with a previously tested and standardized instrument. The PNS project was approved by the National Research Ethics Commission (CONEP) under protocol number 10853812.7.0000.0008, on June 26, 2013. All participants signed the informed consent form.

### Measures

The PNS questionnaire was divided into three parts: household composition; information related to all the household residents; and individual information. The topics covered in each part of the PNS questionnaire have been published elsewhere (18).

All variables used in this study were self-reported. The outcome was created from a variable used to evaluate experiences of discrimination related to health services, based on the following question: “Have you ever felt discriminated against by a physician or another health professional, or have you received worse treatment than others in the healthcare services, regarding one of these reasons?” With yes or no response options, the motivations for discrimination were: Social status (Lack of money, Low social class, Type of occupation), Racial status (Race/color), Type of disease (visually identified), Sex, Age and others (Sexual preference, Religion/belief, any other).

The sociodemographic section included: I) sex (male and female); II) age (in years, categorized in groups from 18–29; 30–44; 45–64; >65); III) color/race (white, brown, black, yellow and indigenous); IV) marital status (married/living together, separated, widowed, single); V) education (illiterate, elementary education or high school, university or postgraduate).

The questionnaire included a module on dental characteristics. This included the self-reported number of upper and lower teeth, which were investigated by the following question: “Regarding your upper (lower) teeth, have you lost any?” The response options were “No,” “Yes, I lost all my upper (lower) teeth,” “Yes, I lost some teeth.” In the case of the last response, the interviewee was asked the number of missing teeth, with answer options from 1 to 15. Data on the use of complete upper and lower prostheses were also collected, using the question “Do you use any type of dental prosthesis (artificial tooth)?”

### Statistical Analyses

Analyses of the absolute and relative frequencies were conducted. The statistical analysis evaluated the association between discrimination in health services, discrimination motivation and sociodemographic and dental variables by means of the chi-square test for linear trends in cases of ordinal variables or heterogeneity in case of categorical variables. The analysis was performed by means of Poisson regression with robust variance to obtain the crude and adjusted estimates of prevalence Ratios (PR) and their respective 95% confidence intervals (CI). The adjusted model included all sociodemographic and dental variables. The analyses were carried out using SPSS v.21 software (Chicago: SPSS Inc.).

## Results

A total of 60,200 subjects were interviewed. The non-response rate of the overall survey of participants was 8.1%. The majority of the participants of the PNS study were female (34,280; 56.9%), in the 30–44 age group (20,242; 33.6%), brown (29,511; 49%), single (27,026; 44.9%) and with elementary or secondary education (45,737; 76.0%). Regarding oral health conditions, most of the respondents had all the teeth in the upper arch (22,376; 37.2%) and had lost from one to four teeth in the lower arch (24,962; 41.5%). The prevalence of self-reported discrimination in health services (the outcome) was 11.5%.

When stratified by the study outcome (discrimination in health services), it was observed that the most individuals who self-reported experiencing discrimination were women (4,228; 12.3%), in the 30–44 age group (2,591; 12.8%), black (778; 13.8%), marital status separated (666; 14.1%), and illiterate (753; 12.8%). Regarding oral health conditions, individuals with dental loss in the upper arch had lost 9–15 teeth (334; 13.9%), and those with dental loss in the lower arch had lost five to eight teeth (648; 14.1%). All sociodemographic variables and dental characteristics are significantly associated with discrimination in health services (*p* < 0.001) (Table 1).

**TABLE 1:** Proportion of discrimination in health services by sociodemographics and dental characteristics.

Characteristics		Discriminated in health services					
		Total	Yes		No		*p*-value^a^
			%	n	%	n	
Sex	Male	25,920	10.5	2,710	89.5	23,210	<0.001
	Female	34,280	12.3	4,228	87.7	30,052	
Age	18–29	14,320	10.3	1,473	89.7	12,847	<0.001
	30–44	20,242	12.8	2,591	87.2	17,651	
	45–64	17,926	12.2	2,193	87.8	15,733	
	<65	7,712	8.8	681	91.2	7,031	
Color/Race	White	24,105	10.1	2,434	89.9	21,671	<0.001
	Brown	29,511	12.2	3,595	87.8	25,916	
	Black	5,631	13.8	778	86.2	4,853	
	Yellow	533	11.4	61	88.6	472	
	Indigenous	420	16.7	70	83.3	350	
Marital status	Married/living together	23,739	11.2	2,650	88.8	21,089	<0.001
	Separate	4,727	14.1	666	85.9	4,061	
	Widowed	4,708	10.1	477	89.9	4,231	
	Single	27,026	11.6	3,145	88.4	23,881	
Education	Illiterate	5,867	12.8	753	87.2	5,114	<0.001
	Elementary education or high school	45,737	11.7	5,351	88.3	40,386	
	University or postgraduate	8,596	9.7	834	90.3	7,762	
Upper number of teeth	All teeth	22,376	9.6	2,148	90.4	20,228	<0.001
	1–4 lost teeth	20,197	12.8	2,577	87.2	17,620	
	5–8 lost teeth	4,204	13.7	574	86.3	3,630	
	9–15 lost teeth	2,403	13.9	334	86.1	2,069	
	Edentulous without prosthesis	2,012	11.8	237	88.2	1,775	
	Complete prosthesis wearing	9,008	11.9	1,068	88.1	7,940	
Lower number of teeth	All teeth	21,115	9.2	1,940	90.8	19,175	<0.001
	1–4 lost teeth	24,962	13.0	3,238	87.0	21,724	
	5–8 lost teeth	4,598	14.1	648	85.9	3,950	
	9–15 lost teeth	2,559	13.6	349	86.4	2,210	
	Edentulous without prosthesis	1,872	10.8	203	89.2	1,669	
	Complete prosthesis wearing	5,094	11.0	560	89.0	4,534	
Total		60,200	11.5	6,938	88.5	53,262	

Brazil, 2019.

a Chi-square test.

Table 2 presents an adjusted analysis regarding discrimination in health services and social and dental characteristics separated by sex. Women presented a higher prevalence of discrimination in the age range between 30 and 44 years (PR = 1.09, 95% CI 1.01–1.18), with brown color/race (PR = 1.09, 95% CI 1.02–1.16), with black color/race (RP = 1.32, 95% CI 1.20–1.46), with an indigenous background (PR = 1.61, 95%, C, 1.25–2.09), and with separated marital status (PR = 1.31, 95% CI 1.19–1.44). Regarding dental characteristics, the higher the tooth loss in the upper arch was, the higher the prevalence of discrimination was: one to four lost teeth (PR = 1.43, 95% CI 1.27–1.62), five to eight lost teeth (PR = 1.75, 95% CI, 1.28–2.39), 9–15 lost teeth (RP = 1.91, 95% CI 1.01–3.14) and lost teeth without dental prosthesis (PR = 2.41, 95% CI 1.18–4.92). In the lower arch, there was a higher prevalence of discrimination in women with one to four lost teeth (PR = 1.53, 95% CI 1.38–1.70) and with five to eight lost teeth (PR = 1.65, 95% CI 1.07–2.79). On the other hand, a protective factor regarding the prevalence of discrimination was observed with the increase in age and in schooling. For men, a higher prevalence of discrimination was found in those with brown color/race (PR = 1.18, 95% CI 1.09–1.29) and black color/race (RP = 1.24, 95% CI 1.10–1.41), as well as in those with higher tooth loss in the upper arch: one to four lost teeth (PR = 1.36, 95% CI 1.17–1.57), five to eight lost teeth (PR = 1.40, 95% CI, 1.16–1.77), 9–15 lost teeth (RP = 2.18, 95% CI 1.25–3.82) and with lost teeth without dental prosthesis (PR = 3.56, 95% CI 1.48–8.60). In the lower arch, there was a higher prevalence of discrimination in men with one to four lost teeth (PR = 1.41, 95% CI 1.24–1.61) and with five to eight lost teeth (PR = 1.40, 95% CI 1.16–1.77).

**TABLE 2:** Unadjusted and adjusted analysis by Poisson Regression (PR) between discrimination in health services and social and dental characteristics, separeted by sex.

Characteristics		Male		Female	
		Adjusted^a^ PR		Adjusted^a^ PR	
		PR	95% CI	PR	95% CI
Age	18–29	1	—	1	—
	30–44	1.07	0.95–1.18	1.09	1.01–1.18
	45–64	0.85	0.75–0.96	0.91	0.82–1.01
	<65	0.65	0.54–0.77	0.61	0.53–0.71
Color/race	White	1	—	1	—
	Brown	1.18	1.09–1.29	1.09	1.02–1.16
	Black	1.24	1.10–1.41	1.32	1.20–1.46
	Yellow	0.82	0.50–1.34	1.24	0.95–1.63
	Indigenous	1.23	0.82–1.84	1.61	1.25–2.09
Marital status	Married/living together	1	—	1	—
	Separate	1.11	0.97–1.28	1.31	1.19–1.44
	Widowed	1.02	0.83–1.26	0.97	0.87–1.09
	Single	0.98	0.91–1.07	1.03	0.96–1.10
Education	Illiterate	1	—	1	—
	Elementary education or high school	0.88	0.78–0.98	0.96	0.86–1.06
	University or postgraduate	0.79	0.67–0.92	0.86	0.78–0.99
Currently smoking	No	1	—	1	—
	Yes	1.14	1.04–1.24	1.26	1.16–1.37
Upper number of teeth	All teeth	1	—	1	—
	1–4 lost teeth	1.36	1.17–1.57	1.43	1.27–1.62
	5–8 lost teeth	1.40	1.16–1.77	1.75	1.28–2.39
	9–15 lost teeth	2.18	1.25–3.82	1.91	1.01–3.14
	Edentulous without prosthesis	3.56	1.48–8.60	2.41	1.18–4.92
	Complete prosthesis wearing	1.36	0.82–2.25	1.26	0.86–1.89
Lower number of teeth	All teeth	1	—	1	—
	1–4 lost teeth	1.41	1.24–1.61	1.53	1.38–1.70
	5–8 lost teeth	2.10	1.19–3.72	1.65	1.07–2.79
	9–15 lost teeth	2.20	0.39–12.4	1.48	0.23–9.36
	Edentulous without prosthesis	1.68	0.48–6.43	3.99	0.40–8.42
	Complete prosthesis wearing	2.97	0.59–14.3	1.80	0.66–4.83

Brazil, 2019. PR, Prevalence Ratio.

a All variables in the table included in the adjusted model.

The proportion of respondents experiencing discrimination in health services due to dental appearance when stratified by motivation shows that there was a significant association between the number of teeth in the upper arch and the number of teeth in the lower arch with social status (*p* < 0.001), type of disease (*p* < 0.001), age (*p* < 0.001) and others, namely, sexual preference (*p* = 0.005) and religion (*p* < 0.001) (Table 3).

**TABLE 3:** Proportion of discrimination in health services by dental appearance, stratified by motivation.

Dental characteristic		Racial		Social status		Type of disease		Sex		Age		Others	
		Yes n (%)	*p*-value^a^	Yes n (%)	*p*-value^a^	Yes n (%)	*p*-value^a^	Yes n (%)	*p*-value^a^	Yes n (%)	*p*-value^a^	Yes n (%)	*p*-value^a^
Upper number of teeth	All teeth	330 (1.5)	<0.001	1,510 (6.7)	<0.001	294 (1.3)	<0.001	101 (0.5)	0.707	193 (0.9)	<0.001	536 (2.4)	0.005
	1–4 lost teeth	442 (2.2)		1968 (9.7)		358 (1.8)		75 (0.4)		274 (1.4)		550 (2.7)	
	5–8 lost teeth	111 (2.6)		446 (10.6)		112 (2.7)		18 (0.4)		88 (2.1)		97 (2.3)	
	9–15 lost teeth	39 (1.6)		243 (10.1)		67 (2.8)		8 (0.3)		53 (2.2)		67 (2.8)	
	Edentulous^b^	36 (1.8)		160 (8.0)		59 (2.9)		8 (0.4)		60 (3.0)		44 (2.2)	
	Prosthesis^c^	103 (1.1)		773 (8.6)		175 (1.9)		31 (0.3)		243 (2.7)		179 (2.0)	
Lower number of teeth	All teeth	315 (1.5)	<0.001	1,375 (6.5)	<0.001	256 (1.2)	<0.001	84 (0.4)	0.689	195 (0.9)	<0.001	455 (2.2)	<0.001
	1–4 lost teeth	528 (2.1)		2,461 (9.9)		475 (1.9)		108 (0.4)		334 (1.3)		713 (2.9)	
	5–8 lost teeth	103 (2.2)		488 (10.6)		128 (2.8)		20 (0.4)		107 (2.3)		122 (2.7)	
	9–15 lost teeth	34 (1.3)		249 (9.7)		68 (2.7)		7 (0.3)		65 (2.5)		58 (2.3)	
	Edentulous^b^	30 (1.6)		136 (7.3)		52 (2.8)		6 (0.3)		66 (3.5)		40 (2.1)	
	Prosthesis^c^	51 (1.0)		391 (7.7)		86 (1.7)		16 (0.3)		144 (2.8)		85 (1.7)	
Total		1,061 (1.8)		5,100 (8.5)		1,065 (1.8)		241 (0.4)		911 (1.5)		1,473 (2.4)	

Brazil, 2019; Others = Sexual preferences and religion.

a Chi-square test.

b Edentulous without prosthesis.

c Complete prosthesis wearing.

As shown in Table 4, there was a higher prevalence of discrimination associated with tooth loss in both the upper and lower arches in all categories observed motivated by social reasons than by other motivations. However, it should be noted that the results for all motivations follow the same direction. There was a higher prevalence of socially-motivated discrimination in edentulous individuals who had tooth loss in the upper arch and who did not wear prostheses (PR = 3.71, CI 95% 2.02–6.79) and in individuals with five to eight lost teeth in the lower arch (PR = 1.93, IC 95% 1.22–3.07).

**TABLE 4:** Prevalence ratio (PR) of reporting discrimination in health services by dental appearance, stratified by motivation.

Dental characteristic		Racial		Social status		Type of disease		Sex		Age		Others	
		PR	95% CI	PR	95% CI	PR	95% CI	PR	95% CI	PR	95% CI	PR	95% CI
Upper number of teeth	All teeth	1	—	1	—	1	—	1	—	1	—	1	—
	1–4 lost teeth	1.26	1.07–1.48	1.56	1.39–1.74	1.10	0.92–1.32	0.76	0.53–1.08	1.39	1.23–1.72	1.06	0.92–1.21
	5–8 lost teeth	1.46	1.13–1.87	1.65	1.21–2.26	1.45	1.12–1.88	0.90	0.4–1.65	1.63	1.21–2.20	0.95	0.74–1.22
	9–15 lost teeth	0.99	0.68–1.46	1.97	1.19–3.26	1.46	1.05–2.02	0.84	0.36–1.96	1.42	0.97–2.08	1.29	0.95–1.75
	Edentulous^a^	1.11	0.70–1.75	3.71	2.02–6.79	1.49	1.04–2.13	1.15	0.45–2.96	1.45	0.97–2.16	1.15	0.78–1.67
	Prosthesis^b^	0.88	0.63–1.23	1.50	1.05–2.14	1.21	0.90–1.62	0.89	0.45–1.77	1.50	1.08–2.08	1.05	0.80–1.38
Lower number of teeth	All teeth	1	—	1	—	1	—	1	—	1	—	1	—
	1–4 lost teeth	1.15	0.98–1.34	1.59	1.45–1.76	1.39	1.27–1.66	1.17	0.83–1.64	1.17	0.96–1.42	1.38	1.21–1.57
	5–8 lost teeth	1.27	0.98–1.65	1.93	1.22–3.07	1.77	1.36–2.30	1.21	0.64–2.30	1.34	1.01–1.81	1.50	1.16–1.92
	9–15 lost teeth	0.90	0.60–1.36	2.68	0.76–9.45	1.66	1.18–2.34	0.77	0.30–2.00	1.28	0.89–1.83	1.33	0.95–1.87
	Edentulous^a^	1.19	0.73–1.93	1.52	0.24–9.46	1.82	1.24–2.66	0.88	0.32–2.46	1.46	0.99–2.17	1.60	1.07–2.38
	Prosthesis^b^	0.92	0.60–1.40	1.48	0.41–5.31	1.27	0.88–1.82	0.89	0.40–1.98	1.19	0.84–1.69	1.16	0.82–1.63

Brazil, 2019.

a Edentulous without prosthesis.

b Complete prosthesis wearing.

## Discussion

This is an original nationwide study that evidenced the existence of social discrimination regarding dental aesthetic conditions based on self-reported information by users of health services in Brazil. The results of this study reveal a type of discrimination in Brazilian culture and identify an association between tooth loss and the self-perception of discrimination in health services. This finding means that the absence of any tooth can create an atmosphere that leads the patient to sense discrimination. Higher self-reported social discrimination was associated with independent variables such as color/race, sex, and illiteracy. In other words, there is a higher prevalence of discrimination towards young black women and those who are illiterate. Added to this, discrimination within the healthcare services affects people who are socially disadvantaged due to ethnicity, immigration status, and religion (22). It is important to highlight that social discrimination is a significant contributor to negative health outcomes regarding minority populations (23) and that the use of dental services is higher among individuals with higher income (24).

This is the first national study in Brazil that considers the association between tooth loss condition and social discrimination when accessing the public health system. This is an original study that reveals important social factors that may lead to discrimination when individuals are seeking health assistance. Studies have shown important factors that lead to discrimination. Self-reported racism and lack of money to pay for dental treatment were described by Australian aboriginal women as associated with toothache experiences (25). Patient skin color may influence the dentist to choose a simpler procedure (26). It is important to show that socioeconomic disparities create a collective experience of discrimination in many venues, including in healthcare services (27, 28). In addition, exclusionary treatment reflects limited healthcare services accessibility (29). From another perspective, one study found no difference between male and female smokers regarding dental appearance and social discrimination in healthcare services (12).

Tooth loss, edentulism (16), dental problems, and necessity of dental prosthesis are still major problems in Latin American countries, such as Brazil. Dental condition is directly associated with quality of life and social discrimination in healthcare services (12, 30, 31). Social discrimination associated with facial aesthetic conditions has been widely described in the literature (8, 32, 33). The present study brings up the discussion about the influence of tooth loss and the use of prosthesis on the presence of discrimination in health services. Social judgments are sometimes influenced by dental appearance, which means that the appearance of tooth loss and decay are triggers of discrimination (34). This corroborates the present study findings that the more teeth a person has lost, the higher the chance of being discriminated against in health services.

In addition, recent studies support the idea that discrimination in healthcare services is common (7, 12) and can be considered a public health problem because persons who report discrimination may be less likely to receive preventive health services (35). In this context, it is important to highlight the present results that tooth loss implies a greater prevalence of discrimination. This may happen because of stereotyping in regard to a poor dental appearance (1, 8). Reference 36 demonstrated that edentulous individuals showed a 73% higher probability of not visiting the dentist than individuals who had 10 or more teeth. The present study found that there was a higher prevalence of social discrimination in edentulous individuals who did not wear a prosthesis in the upper arch. However, in edentulous individuals who wore dental prostheses, the use of the prostheses was a protective factor for the studied outcome. These findings have been confirmed in the literature because a prosthesis can nearly replace the facial appearance and minimize the effects of tooth loss by improving interpersonal relationships (37, 38). These results indicate that the dental prosthesis is a strong factor influencing discrimination in healthcare services. Therefore, discriminatory practices may be based on characteristics, such as physical appearance (2).

An important limitation of this study is the measurement of discrimination, since studies should prioritize the definition of a construct map and the simultaneous evaluation of different levels and motivations in the occurrence of discrimination. The joint exploration of these aspects would provide a broader understanding of discriminatory patterns and their consequences for health, thus increasing our ability to reduce their occurrence in society.

This study has several advantages and strengths. The main strength includes its relevant social proposal regarding aspects of oral health. Furthermore, it presents the originality of exploring the association between tooth loss and prejudice, and it adopts a nationally representative sample of health service users. Additional research is needed to investigate the specific reasons for why users perceive discrimination in healthcare services. With these limitations and strengths, this study offers important findings supporting the conclusion that social discrimination associated with the number of teeth and dental prosthesis wearing is a reality in Brazil and needs to be deeply investigated to promote good oral health possible for everyone.

## Data Availability

The original contributions presented in the study are included in the article/Supplementary Material, further inquiries can be directed to the corresponding author.
